# The Challenge of *Global Challenges*

**DOI:** 10.1002/gch2.201870003

**Published:** 2018-01-22

**Authors:** Jörn Ritterbusch, Kirsten Severing, Prisca Henheik, Till von Graberg, Yan Li, Anna Tröger

The new year has just begun, and we are still all full of hope and confidence that it will be a good one for you, my fellow readers, and for the world we are living in. However, when we look back at 2017, it is difficult to be very optimistic. We are not sure about you, but we are not totally convinced that mankind has recently succeeded towards making this planet a better place. Discord and bigotry, in an attempt to gain fast and short‐term profits rather than cooperation and sustainable developments, seem to be back again and even stronger than in previous years, on the agenda of many powerful decision‐makers in policy and economy.

Nevertheless, we are convinced, more than ever, that there is a multitude of engaged scientists out there who know that it is in our mutual interest to address the global challenges that we are facing together. And this confidence in the ability of scientists and researchers to join their efforts to create solutions for our world enabled us to launch the first volume of this new scientific journal, *Global Challenges*. Our vision is to mobilize research, debate, and leadership in the global challenges and create a platform for directing and setting the research and policy agenda. This journal started, initiated by the founding editors, with a clear mission:

- Create a community of researchers, policy makers, practitioners and funders engaged in addressing complex global problems
- Advance research and enable decision makers to base policy and practice on scientific evidence
- Encourage multidisciplinary conversations between scientific fields and between scientists and social scientists
- Articulate the policy and practice implications of primary research
- Make measurable progress in the mitigation of global challenges

As editors we certainly need to question if the papers published were in line with this mission, despite the tough circumstances that every new journal has to face. If we look at the highlights of the first volume, we can certainly be happy about our achievements. Several articles are cited already multiple times, we have very good usage numbers, and some papers were taken up by the media quite heavily. **Table** **1** displays the papers that had the biggest impact regarding citations, usage, and Altmetric scores, and from this you can see that our aim of topical diversity, addressing complex global problems, was certainly achieved. Climate change, water resources and management, solar energy, and global health issues are discussed in these highlighted papers.

**Table 1:** Published papers with high citations, usage, and Altmetric values in Volume 1 of Global Challenges.

DOI	Title	Author(s)	Volume	Issue	Cites
10.1002/gch2.201600008	Inoculating the Public against Misinformation about Climate Change	Sander van der Linden, Anthony Leiserowitz, Seth Rosenthal and Edward Maibach	1	2	16
10.1002/gch2.1018	Data, disease and diplomacy: GISAID's innovative contribution to global health	Stefan Elbe and Gemma Buckland‐Merrett	1	1	3
10.1002/gch2.201600003	Extremely cost‐effective and efficient solar vapor generation under non‐concentrated illumination using thermally isolated black paper	Zhejun Liu, Haomin Song, Dengxin Ji, Chenyu Li, Alec Cheney, Youhai Liu, Nan Zhang, Xie Zeng, Borui Chen, Jun Gao, Yuesheng Li, Xiang Liu, Diana Aga, Suhua Jiang, Zongfu Yu and Qiaoqiang Gan	1	2	16
10.1002/gch2.1010	Reliable, resilient and sustainable water management: the Safe & SuRe approach	David Butler, Sarah Ward, Chris Sweetapple, Maryam Astaraie‐Imani, Kegong Diao, Raziyeh Farmani and Guangtao Fu	1	1	3
10.1002/gch2.1022	Global health governance in the sustainable development goals: Is it grounded in the right to health?	Remco Van de Pas, Peter S. Hill, Rachel Hammonds, Gorik Ooms, Lisa Forman, Attiya Waris, Claire E. Brolan, Martin McKee and Devi Sridhar	1	1	3


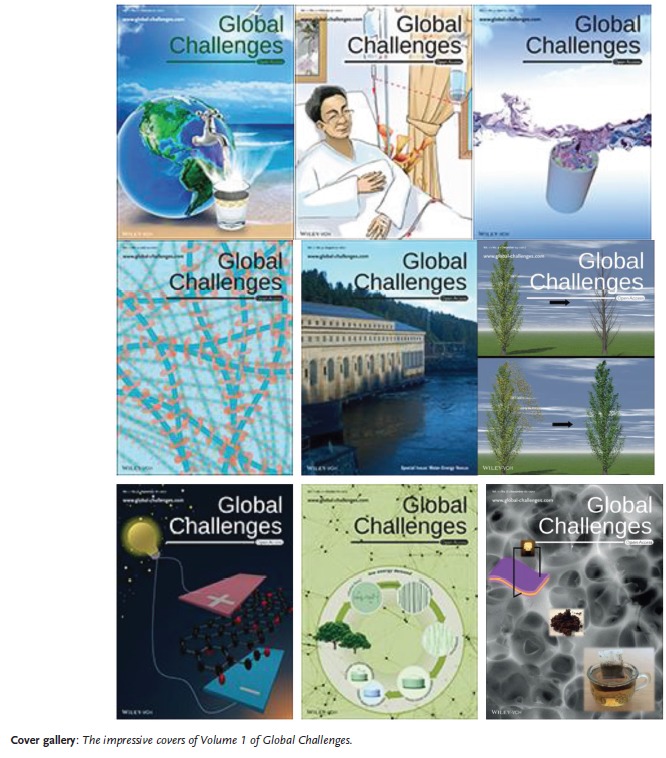


Apart from publishing exciting science, one of the first milestones in the history of a young journal is its inclusion in major indexing services. We are happy to announce that *Global Challenges* has been accepted for inclusion in *Web of Science (the Emerging Sources Citation Index*), which is an important step for increased discoverability and accessibility.

With many ideas in mind and fantastic feedback from our board members, authors, and reviewers, we are very much looking forward to further develop *Global Challenges* after its very promising start. Thank you to all of those who made this journal a success so far and don't miss out on the chance to gain access to the next volume of *Global Challenges*. It's open to everyone!

